# Supplementary material for: Assessment of pharmacogenomic agreement
Source: F1000Res. 2016 May 9;5:825. [Version 1] doi: 10.12688/f1000research.8705.1 (PMC4926729; doi:10.12688/f1000research.8705.1)
Supplement: Peer review history

# Peer review: Referee response for version 1

**DOI:** 10.5256/f1000research.9367.r14152

**Published:** 2016-06-29

**Authors:** Yudi Pawitan

**Affiliations:** 1 Department of Medical Epidemiology and Biostatistics, Karolinska Institute, Stockholm, Sweden

The paper highlights the curious lack of rigorous standards for what constitutes ‘agreement’, ‘consistency’ between genomic studies, or more generally, the fundamental issues of ‘validation’ and ‘reproducibility’, etc. The problem is even more serious of results based on high-throughput omics data as the potential for false positive is substantial.

 The persistent lack of consensus or standards may partly indicate that these issues are not so straightforward. The main problem is that when we say we ‘validate’ a result, this can be done at different strengths. For example, consider the commonly performed method in statistical analyses, the so-called ‘cross-validation’, where we split our total sample into training and validation sets. If the split is done randomly, then we have only a ‘soft validation’, since it applies to the same sample (or same lab, same population, same measurement method, etc) so the ‘validation’ is internal and corresponds to statistical significance only. In contrast a scientist may wish for something stronger,  for an external validation, for example, for the ‘biological truth’ to apply other populations; thus, one study may be performed in a European population, but the external validation is done in an Asian population. The latter is a stronger validation than the random-split validation, giving a more compelling and general biological story. What is relevant here is that both validations are commonly done in practice, and both are valid, but they carry different levels of information. I think what matters in practice is that the implication of the validation should always be clear (or clarified), so that the user of the information can judge its relevance.

 The key point of Safikhani *et al *is that their 2013 validation study of the genomic predictors of drug-sensitivity was more stringent than the 2015 validation studies by the GDSC and CCLE investigators. This is clearly highlighted in Figure 1, where the latter used the same molecular data, so the ‘validation’ is only of the pharmacological data and perhaps (not clear to me) the method of analyses. Which level of validation is more relevant here? Let us imagine how the results (eg the genomic predictors) are to be used in patients. The molecular data are likely to be generated and analyzed in a diversity of labs, so the genomic predictors should really be robust to the actual heterogeneity in the molecular data. The results (the genomic predictors) may not survive such stringent requirements, but that is what we need to know. So, overall, I agree with Safikhani *et al *that a more stringent validation allowing for variability in both molecular and pharmacological data is more relevant in this context of drug prediction.

 (However, reading Haibe-Kains *et al*, there seemed to be an emphasis that the failure of agreement was due to the high variability in the pharmacological data. So it is possible that the later studies by the GDSC-CCLE investigators responded to this concern only.)

 Regarding specific issues in the paper:

1. I do not consider the use of most recent data as a key issue.
2. I agree that the choice of IC _50_ in GDSC vs AUC in CCLE is puzzling and only raises a question mark regarding the results.
3. Arbitrary cutoffs in defining what constitutes an ‘agreement’ are unnecessary if authors can refrain from using judgmental  words like ‘high consistency’ etc., especially when used as a summary statement across distinct drugs. It would be better to just report the actual performance for each drug or for each cancer type, since it is still not clear how these statistics would translate in terms of clinical cost-benefit balance.

I have read this submission. I believe that I have an appropriate level of expertise to confirm that it is of an acceptable scientific standard.

# Peer review: Referee response for version 1

**DOI:** 10.5256/f1000research.9367.r14596

**Published:** 2016-06-27

**Authors:** Terence Speed

**Affiliations:** 1 Bioinformatics division, Walter and Eliza Hall Institute of Medical Research, Parkville, Vic, Australia

I found the title appropriate, and that the abstract represented a suitable summary of the work.

 I believe that the design, methods and analysis of results are appropriate for the topic being studied, and that for the most part, they were clearly explained. A couple of perceived shortcomings are itemized here.

 p.3, column 2, line 2. The “but” would be better replaced by “and”.

 p.5. Figure 2. The dotted and solid diagonal lines on these plots are not identified in either the caption or the text.

 p.5, Figure 3. It is nowhere explained whose Pearson correlations (PCC) are summarized in these box plots. I suppose that some number (to be stated) of cell lines were profiled in both GDSC and CCLE, and that in all cases, the PCC in the box plots are calculated from molecular data from pairs consisting of the data on the same cell line generated in GDSC and in CCLE. A clear statement along these lines would be helpful.

 p.6, column 1, lines 1-4. This assertion would have more force if the authors told the reader how many cell lines *could* have contributed PCC to the box plot of Figure 3a, and how many *did* do so.

 Further, I do believe that the conclusions are sensible, balanced and justified on the basis of the results of the study.

 Finally, I understand that all the data used in this study is available, and this is also true for the code used to generate all the results and figures.

I have read this submission. I believe that I have an appropriate level of expertise to confirm that it is of an acceptable scientific standard.

# Peer review: Referee response for version 1

**DOI:** 10.5256/f1000research.9367.r14316

**Published:** 2016-06-10

**Authors:** Weida Tong

**Affiliations:** 1 Division of Bioinformatics and Biostatistics, National Center for Toxicological Research, U.S. Food and Drug Administration, Jefferson, AR, USA

It is a lot to take/digest the manuscript. I break this story into three parts:

- In 2012, both GDSC and CCLE released/published drug sensitivity data (both pharmacological and genomic). In 2013, the authors compared the two studies using the drugs in common between two. Their analysis was carried out in a direct fashion which account for variations of both genomic and pharmacological data from the same site (GDSC or CCLE) and found the results between two did not agree.
- Recently, GDSC/CCLE did an independent analysis and demonstrated that the agreement between two are actually higher (using ANOVA) than what the authors reported. They concluded that the results between GDSC and CCLE were consistent. However, the comparison was only focused on the pharmacological data because the genomic data used actually came from one site. That means their analysis did not include the noise introduced by both sites in this comparison.
- The authors, again, reanalyzed data by including pharmacological and genomic data from both sites and the conclusions remain as the same as they reported in 2013.

I have no problem with their analysis and support their conclusions. With that said, I did find the paper could flow better by moving two sections into Discussion. These are:

- “Which pharmacological drug response data should one use?” - It seems odd and smell bad that GDSC/CCLE used the data published in 2012 and totally ignored the most current data in their analysis. This could be due to many different reasons. Thus, speculation is not necessary considered as “results”. I would say this will be better justified as “discussion”.
- “What constitutes agreement” – Again, this is a difficult call. I believe there is no single baseline that can be used to justify consistency. Thus, most text in this section will sit better in “discussion”.

Overall, I support its indexation with revision by focusing on the flow of the story and the structure of manuscript.       

I have read this submission. I believe that I have an appropriate level of expertise to confirm that it is of an acceptable scientific standard.
